# Treatment of Asphyxia from Drowning

**Published:** 1842-03

**Authors:** 


*Treatment of Asphyxia from drowning.*—As most practitioners in the city or country are occasionally called upon to attempt the resuscitation of persons apparently drowned, yet not so frequently as to bear in mind at all times the differences of opinion among the more experienced, as to the artificial respiration, and the time at which it becomes useless, we extract the following remarks from a debate on the subject before the Westminster Medical Society, in October last, as published in the London Lancet for October 30th.

Mr. Forbes Winslow led off this portion of the debate, which was collateral with a discussion on the various forms of asphyxia in new-born infants, eliciting nothing new, as far as we can judge from the report.

"Mr. Winslow was of opinion that inflation by air breathed from the mouth of the practitioner was the best plan of carrying on artificial respiration. Experiments had shown that the air so used was but little altered in quality, and contained only one-hundredth part less of oxygen than it did previous to its being respired. With respect to the recovery from asphyxia by drowning, Dr. A. T. Thomson had related a case in which a person who had been twenty minutes under water was restored; while other cases were recorded, in which three minutes' immersion was sufficient to entirely destroy life, and frustrate all efforts for its revival. How did we account for this discrepancy? The most experienced pearl-divers could not remain under water more than five minutes. He

(Mr. Winslow) thought that when persons fainted immediately on submersion, vitality was longer retained in consequence of there being less demand upon the system than when struggles were made by the sufferer.

"Dr. Chowne would look with suspicion on any case of recovery from asphyxia by drowning, in which it was stated that the person had been under water for more than six or seven minutes. He believed, indeed, that there was no well-authenticated case of recovery in which the submersion had exceeded six minutes."

"Mr. Wooley thought Mr Read's instrument would be of great service in asphyxia, whether occurring in adults or infants. He had been in the habit of using the means recommended by the Humane Society to keep up artificial respiration, for some years. The instruments used were the bellows and the trachea-tube. The use of the bellows might be productive of much injury; and it was not always an easy matter, even to those who knew the anatomy of the parts well, to insert the trachea-tube into the proper orifice. Mr. Read's instrument did away with these objections, and he considered it an admirable means of keeping up artificial respiration." "In asphyxia from drowning, however, he had seldom found much benefit from attempts to keep up artificial respiration. When a body was brought to the receiving-house, it was immediately placed in a bath at 100°. Respiration most frequently occurred spontaneously whilst the person was in the bath, and in some cases it took place before the patient had arrived at the station. If, after the body had been placed in the bath, there was no appearance of vitality, he had generally found the employment of artificial respiration of no benefit. Respiration, when first returning, was generally interrupted and spasmodic; it then became regular, and frequently no ill consequences followed. There was, however, in many cases, much mischief supervening in the form of congestion of the brain and thoracic viscera. In some cases of this kind, the convulsions were so great, that the patient was held with difficulty whilst blood was abstracted. In these cases the only instrument he had found of service was the lancet; and it was often necessary to take away a considerable quantity of blood."

Mr. Wooley then read a case illustrating the length of time during which life may be preserved. It was first published in the report of the Royal Humane Society in 1840, but as the report is in few hands in America, we extract from it every thing essential.

"At about half-past ten in the morning, a police-constable on duty, and the gate-keeper, saw a man, a little more than half way over the bridge, place his hat upon the pavement, mount the parapet, and leap into the water. The policeman ran to the spot where the occurrence had taken place, and the gate-keeper to his lodge, in which was one of the society's speaking-trumpets, to use it in calling for a boat; but before doing so, he took out his watch, and carefully observed the time. When he saw the boat coming, he ran and joined the policeman and others who were observing the man in the water. From the time of his jumping in he had remained under the surface, supported in a perpendicular position by the flaps of his coat, which lay *upon* the surface, a small portion of the scalp being out of the water. His face was never above the surface, and a great number of small air-bubbles kept coming up from the commencement. He moved very little—at length a large bubble came up, and he immediately began to sink deeper; the bystanders now exclaimed that it was all over with him. At this moment the boat reached the spot, when he had sunk so low that his coat tails were but just within the reach of the arm of the man employed by the institution, who, however, got hold of them, and pulled him into the boat. The gate-keeper then looked at his watch, and found that exactly *five minutes* had elapsed since he first noted the time. The man was apparently dead; but just after he was laid down in the boat a low moan was heard, another midway between the bridge and the receiving-house, and a third when he was on the stretcher being carried into the house. There was, however, no *appearance* of breathing or of life when he was put into the warm bath, which was ready upon his arrival; but, almost immediately after, convulsive and irregular breathing commenced—he breathed irregularly and moaned occasionally for about fifteen minutes. In five minutes more I saw him; his breathing was then quite natural, his pulse regular and good; he had no pain in the head or chest, nor any where else, and felt pretty well; his face, however, was very pallid, and his eyes suffused: he looked like a man who had suffered from habitual intemperance, which I found was the case. I now had him taken from the bath, and put upon the warm bed, apparently free from cerebral or *thoracic* congestion, and only wanting strength to enable him to go home. As the ward was too hot and close, I desired that a little air might be let in, and left the room for a short time. On my return, I found that my order had been misunderstood, and all the windows had been opened. I was absent only a very few minutes in the adjoining room. The effect of this admission of cold air was to produce oppressed breathing and much cough, and to require treatment and watching, which detained me six hours at the receiving-house; they would not take him into St. George's Hospital, and, till the expiration of that time, I did not think it safe to send him home. This shows the importance of warm air in resuscitation."—*Philad. Examiner.*

